# Automatic optimization of regions of interest in hyperspectral images for detecting vegetative indices in soybeans

**DOI:** 10.3389/fpls.2025.1511646

**Published:** 2025-03-06

**Authors:** Sangyeab Lee, Amit Ghimire, Yoonha Kim, Jeong-Dong Lee

**Affiliations:** ^1^ Department of Applied Biosciences, Kyungpook National University, Daegu, Republic of Korea; ^2^ Department of Integrative Biology, Kyungpook National University, Daegu, Republic of Korea; ^3^ Upland Field Machinery Research Center, Kyungpook National University, Daegu, Republic of Korea

**Keywords:** vegetative indices (VIs), hyperspectral imaging, normalized difference vegetation index (NDVI), photochemical reflectance index (PRI), anthocyanin reflectance index (ARI)

## Abstract

Vegetative indices (VIs) are widely used in high-throughput phenotyping (HTP) for the assessment of plant growth conditions; however, a range of VIs among diverse soybeans is still an unexplored research area. For this reason, we investigated a range of four major VIs: normalized difference vegetation index (NDVI), photochemical reflectance index (PRI), anthocyanin reflectance index (ARI), and change to carotenoid reflectance index (CRI) in diverse soybean accessions. Furthermore, we ensured the correct positioning of the region of interest (ROI) on the soybean leaf and clarified the effect of choosing different ROI sizes. We also developed a Python algorithm for ROI selection and automatic VIs calculation. According to our results, each VI showed diverse ranges (NDVI: 0.60–0.84, PRI: −0.03 to 0.05, ARI: −0.84 to 0.85, CRI: 2.78–9.78) in two different growth stages. The size of pixels in ROI selection did not show any significant difference. In contrast, the shaded part and the petiole part had significant differences compared with the non-shaded and tip, side, and center of the leaf, respectively. In the case of the Python algorithm, algorithm-derived VIs showed a high correlation with the ENVI software-derived value: NDVI −0.97, PRI −0.96, ARI −0.98, and CRI −0.99. Moreover, the average error was detected to be less than 2.5% in all these VIs than in ENVI.

## 1 Introduction

Plant phenotyping involves the process of acquiring phenotypic data (observable traits or characteristics of any organism) throughout the developmental stages of the crop plant at different scales with analysis of multidimensional phenotypes in an accurate and precise manner (Yang et al., 2020). The advancement of analytical techniques, reduced data collection durations, increased automation, and the ability to acquire large amounts of data with a high level of precision and thoroughness have led to the emergence of the plant phenotyping technique known as high-throughput phenotyping (HTP) (Kim et al., 2020b). At present, HTP based on imaging technique has been a topic of research interest, which has led to the acquisition of data based on image sensors more accurately, non-invasively, and automatically; thus, it has been extensively utilized for the assessment of the quantitative and qualitative characteristics in crops (Li et al., 2014, 2018; Barboza et al., 2023). Various imaging techniques like red, green, and blue (RGB) imaging/visible imaging, fluorescence imaging, thermal imaging, tomography (magnetic resonance imaging, positron emission tomography, computed tomography scanning), and spectral imaging have been widely used for phenotyping of different plants (Fiorani and Schurr, 2013; Omari et al., 2020). Among them, the hyperspectral imaging (HSI) system has the capacity to extract both structural and physiological data from plants concurrently and has the ability to effectively, accurately, and easily capture the phenotypic variation of the biotic and abiotic stress in plants both in field and controlled conditions (Neilson et al., 2015; Mishra et al., 2017; Sarić et al., 2022).

The main way to utilize the valuable information obtained from HSI analysis is by computing vegetation indices (VIs), which are derived by comparing the reflectance values of specific wavelength bands and are selected based on their sensitivity to various vegetation characteristics (Sytar et al., 2017; Kang et al., 2019). VIs derived from the calculation of these key wavelength relationships can be used as a tool to monitor and evaluate changes in the physiological properties and overall health of plants throughout their life cycle and provide both quantitative and qualitative data for the plant cover and growth pattern (Thenkabail et al., 2000). The study utilized ENVI V.5.5.3 software (Research System, Inc., USA) to measure vegetation indices (VIs), which were subsequently categorized into seven distinct groups: broadband greenness, narrowband greenness, canopy nitrogen, canopy water content, dry or senescent carbon, leaf pigments, and light use efficiency (https://www.nv5geospatialsoftware.com/docs/BackgroundVegetationIndices.html, accessed on 29 April 2024). Four different VIs used in this study fall under the abovementioned categories. Normalized difference vegetation index (NDVI) is related to the broadband greenness of the leaf, photochemical reflectance index (PRI) is related to light efficiency, and anthocyanin reflectance index (ARI) and carotenoid reflectance index (CRI) are related to leaf pigments. NDVI measures the greenness of the vegetation and plant canopy properties like leaf area index and light interception (Pettorelli, 2013; Pineda et al., 2022). PRI measures changes in the reflectance of light by leaves, particularly in the wavelength range sensitive to carotenoid pigments like xanthophylls, and can be used to detect physiological stress in plants (Peñuelas et al., 1995; Ryu et al., 2020). ARI indicates a correlation with anthocyanin content, and the higher the concentration of anthocyanin in leaves, the more they are associated with stressed plants (Gitelson et al., 2001; Abdulridha et al., 2019), and CRI has a correlation with carotenoid content (Gitelson et al., 2002). VIs are essential tools to measure the plant growth pattern and the change in biophysical and phenological characteristics of the plant over a period of time (Huete et al., 2002). VIs, which are derived from a wide range of spectrum data, are extensively used in plant breeding and precision agriculture via HTP techniques. VIs are used as major parameters for the selection of waterlogging-tolerant and susceptible soybean accessions (Kim et al., 2021), assessment of bacterial disease in soybean (Then et al., 2023), forecasting the yield of maize and soybean (Bolton and Friedl, 2013), quantification of crop characteristics (Kokhan and Vostokov, 2020), predicting the chlorophyll content based on machine learning (Narmilan et al., 2022), and for the early detection of plant disease (Rumpf et al., 2009).

In plants, spectral characteristics vary significantly in leaves, plant individuals, canopies, and colonies. Hence, there is a need for appropriate hyperspectral image analysis techniques that can represent and capture these diverse structural features (Kim et al., 2020a). In spite of the increasing demand for utilizing HSI, related research has an unidentified research area. In this research, we selected the appropriate pixel size for establishing a region of interest (ROI), particularly concerning VIs within the same leaf when annotating different leaf positions. Additionally, we created a Python algorithm to automatically define the ROI in such a way that the ROI falls on the center of the leaf with no shaded part to reduce ROI annotation and VI calculation time.

## 2 Methodology

### 2.1 Plant material

The entire experiment was conducted at Kyungpook National University (Daegu, South Korea). To observe the variation in vegetative indices (VIs) in soybeans, 258 accessions were randomly selected from the Korean cultivated soybean core collection consisting of 430 accessions (Jeong et al., 2019). Among the cultivated soybeans selected, five germplasms: Sehwa, Hosim 2, Pungsannamul, Seonpung, and Williams 82 were further used as check varieties. The 258 accessions were planted in greenhouse conditions with temperatures ranging from a maximum of 30°C to a minimum of 20°C humidity maintained at 75% with a variation of ± 10%, and a day length of around 14h. The check varieties were also grown in plant growth chambers with growth conditions set up as day and night lengths as 14 and 10h with temperatures of 26°C and 23°C, respectively. The humidity was set to 75% ± 5%. In both conditions, horticulture soil was used as soil material consisting of cocopeat 68%, pittMoss 14.73%, perlite 7%, zeolite 4%, rough stone 6%, and pH modifier 0.005. All the accessions were planted in plastic pots (12 cm × 10.5 cm) consisting of three replications and a single pot designated as one replication.

### 2.2 Hyperspectral image acquisition and image processing

Spectral images were taken using a handheld portable hyperspectral camera, Specim IQ (Oulu, Finland, model: WL18 MODGB, firmware version: 2019.05.31.1) with a 99% barium sulfate white reference and based on Specim’s push-broom technology. This spectral camera captures images ranging from 400 to 1000 nm wavelength, which is further divided into 204 bands. The captured image is of 512 × 512 size. The spectral resolution full-width half maximum is 7 nm, and spatial sampling is 512 pix. The HSI was acquired under two halogen lamps (placed at approximately 45° angle to the plant) for the check varieties grown in the chamber, and the other 258 accession’s HSI was acquired in a normal natural lighting condition. The plant-to-lens distance was kept at 100 ± 10 cm and maintained at almost a 90° angle during the experiment. A white reference was used for the calibration and to maintain the focus until the target was highlighted with the maximum amount of orange-colored indicators. The calibration was done on a regular basis during the experiment for more accurate data collection. This camera has both the hyperspectral sensor and the RGB sensor, which gives both the spectral image and the RGB image of the plant. During the entire experiment, the images of the plants grown in the greenhouse were captured early in the morning, around 8 a.m. to 9 a.m. Similarly, for the growth chamber-grown check varieties, the images were captured during the daytime under artificial halogen light conditions. As mentioned earlier, ENVI V.5.5.3 software (Research System, Inc., Redlands, California, USA) was used to process the spectral images obtained. After acquiring the HSI, they were imported into the software, and ROI was manually defined in individual images.

### 2.3 Calculation of VIs

This study analyzed four different types of VIs. These VIs were selected in such a way that we can gain information about broadband greenness, leaf pigments, and light efficiency and fall under the range of our spectral wavelength, that is, from 400 to 1000 nm. These VIs use specific combinations of different wavelengths, as listed in **Table 1**. After selecting ROI from the individual plants, the band math function in ENVI was used to write these formulas. The specific wavelength was selected every time for individual ROI to calculate the specific VIs. The statistics for ROI were obtained, and the mean value of each statistic was kept as the value of VIs for each of the ROI selected. Thus, this process of manual selection of ROI in individual plants and then again specifying the wavelength for each of the ROI, made obtaining VIs tedious and time consuming.

**Table 1:** Vegetative indices (VIs) used in the study.

Vegetation indices	Formula	References
Normalized difference vegetative index (NDVI)	$\frac{\left(R860nm-R650nm\right)}{\left(R860nm+R650nm\right)}$	(Rouse Jr. et al., 1974)
Photochemical reflectance index (PRI)	$\frac{\left(R531nm-R570nm\right)}{\left(R531nm+R570nm\right)}$	(Peñuelas et al., 1995)
Anthocyanin reflectance index (ARI)	$\frac{1}{R550nm}-\frac{1}{R700nm}$	(Gitelson et al., 2001)
Carotenoid reflectance index (CRI)	$\frac{1}{R510nm}-\frac{1}{R550nm}$	(Gitelson et al., 2002)

### 2.4 Comparison of VIs based on ROI size and ROI position in leaf

**Figure 1:**
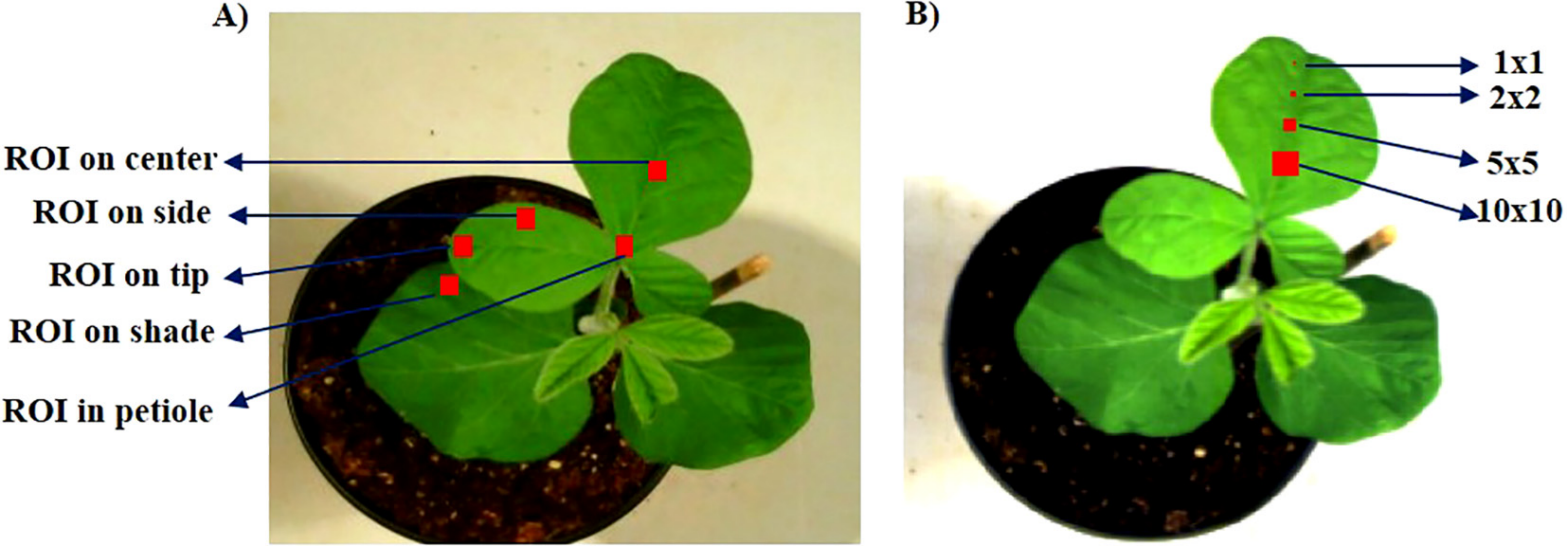
Position of regions of interest (ROIs) in different leaf parts and the comparison of ROI size. **(A)** ROIs in different parts of the same leaf and **(B)** different sizes of ROIs.

To compare the differences within VIs among different pixel sizes of the ROI and the position of the ROI within the same leaf, the HSI of the check varieties were analyzed. To compare the VIs based on ROI position, the leaf was divided into four different regions: center, side, tip, and petiole (**Figure 1A**). We also compared the differences in VIs in the shaded region of the leaf and the non-shaded region. Likewise, to check the effect of ROI size, we annotated the leaf with four different pixel sizes: 1 × 1, 2 × 2, 5 × 5, and 10 × 10 (**Figure 1B**). The comparison was made with the whole leaf annotated in these cases.

### 2.5 Comparison of VIs variation in cultivated soybean

To compare the variation of VIs among cultivated soybean germplasms, 258 accessions were used. In soybeans, the vegetative growth period is divided into different stages depending on the number of trifoliate leaves and nodes in the main stem. When the cotyledon emerges from the soil, it is termed the emergence stage (VE); when unifoliate leaves unfold completely, the stage is termed the VC stage; when the first trifoliate fully opens, it is the first trifoliate stage (V1); when the second trifoliate unfolds fully and plants have three nodes, it is the second trifoliate stage (V2); when the third trifoliate unfolds fully and plants have four nodes, it is the third trifoliate stage (V3); and so on (McWilliams et al., 1999). Similarly, the reproductive growth period is also divided into different growth stages. In this study, HSI was obtained outdoors when each genotype completely reached the VC and V1 stages. Subsequently, NDVI, PRI, ARI, and CRI were calculated using the methods described in **Table 1**.

### 2.6 Automatic ROI selection and VIs calculation

**Figure 2:**
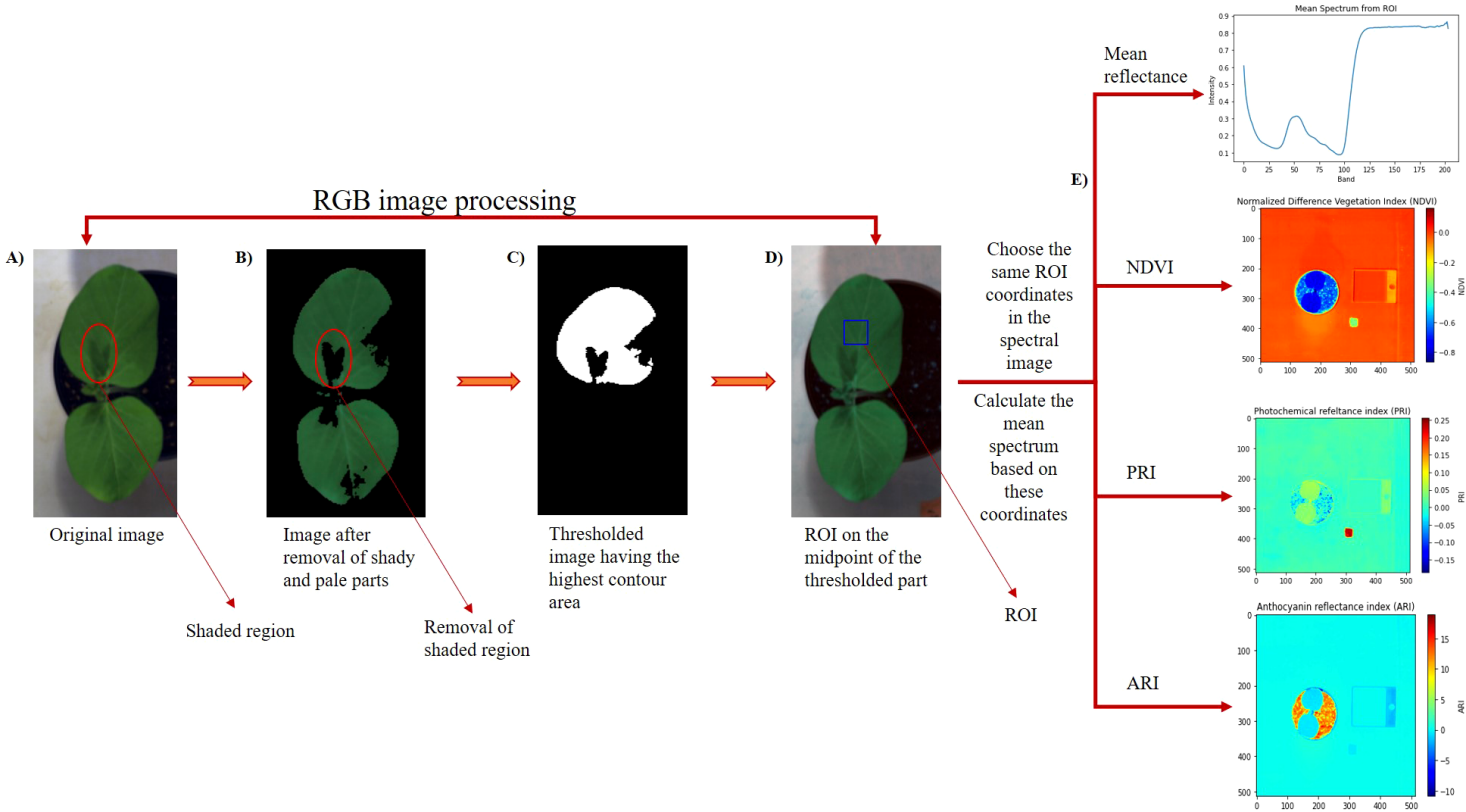
Extraction of region of interest (ROI) and calculation of vegetative indices. **(A)** Whole red, green, and blue (RGB) image, **(B)** image after removal of shaded and pale parts, **(C)** thresholded image, **(D)** RGB image with ROI, and **(E)** spectral image processing.

As mentioned earlier, the manual selection of ROI and specific wavelengths associated with specific VIs was tedious. Thus, we employed a Python algorithm to select ROI and calculate VIs automatically. Python 3.11.4 was used as the coding language, and Spyder IDE 5.5.1 was used as the working environment. The methodology of ROI extraction and VIs calculation has been illustrated in **Figure 2**. The process is divided into two major parts: the first part includes the processing of the RGB image and the second part includes the processing of the spectral image. After importing the RGB image, we defined the upper and lower greenness values for the image (**Figure 2A**). The values represented in defining the upper and the lower greenness level represent the hue, saturation, and value (HSV). The hue indicates the color type, saturation measures the intensity, and value indicates the brightness level. As the shady parts in the leaves have higher intensities and darker than the leaf part with normal lightning conditions, the adjustment in these HSV removes the shady part as well as the background. This generated the leaf image only with normal lighting condition as shown in **Figure 2B**. Furthermore, a manual method of ROI optimization has also been provided in the study where the users can select their own ROI part. This will allow the user to create their own ROI of varying size so that ROI can be adjusted based on the leaf image and user desire. After the removal, the image is then thresholded using two different thresholding methods; Otsu’s threshold and triangle threshold. Then, the thresholded area having the highest contour area was selected (**Figure 2C**). We then found the midpoint of this highest contour area and created an ROI on the midpoint. The ROI plotted is then displayed, and the coordinates of the ROI are extracted (**Figure 2D**). If substantial shaded region are present on the leaf and ROI falls on this shaded region, the size of the ROI can be minimized or adjustment in defining the HSV value can be done. This ensures that the ROI is placed on the region with non-shaded leaf part. The next part begins after the calculation of ROI coordinates, where the work is done in the HSI image. The HSI image is first imported and displayed the image using any one of the bands (as 204 bands are prevalent, any one band can be used to display the spectral image). The ROI coordinates, which were extracted from the RGB image, are then plotted in the spectral image. It must be sure that the image size and orientation in the RGB image match the orientation and image size of the spectral image. Otherwise, the ROI coordinates extracted from the RGB image will not match the same position in the spectral image. The mean spectrum of every band from the plotted ROI is then calculated, and the graph of the mean spectrum is plotted (**Figure 2E**). The formula of each of the VIs is written, and the VIs are plotted along with the value as results (**Figure 2E**). We have provided the source code used during the in (https://github.com/AG9843/ROI-optimization-and-VIs-calculation.git). Apart from automatic ROI selection, manual selection of ROI to calculate the VIs for images containing multiple plants has also been provided. A detailed flowchart of the method is given in **Supplementary Figure S1**. Twenty plants belonging to different growth stages were randomly selected. To confirm the algorithm’s results, the same part of the leaf identified by the automatically generated ROI was manually annotated using ENVI software (**Supplementary Figure S2**). The VIs from that ROI were then calculated. Linear regression, along with the correlation coefficient (*R* ^2^) value, was figured out for the comparison.

### 2.7 Statistical analysis

Statistical analysis was conducted using SAS v9.4 (SAS Institute, Cary, NC, USA, 2013). In an experiment comparing the differences in VIs between the shaded area of the leaf and the part receiving natural light, analysis of variance (ANOVA) with PROC GLM was utilized. Subsequently, multiple comparison analysis was conducted using the least significant difference (LSD) method. For the study on finding the appropriate pixel size and ROI position in the leaf and the exploration of VI variations, a Student’s t-test was conducted using PROC GLM. The analysis of Pearson’s correlation between the four VIs was conducted using RStudio. The linear regression within 95% confidence interval and 95% prediction interval was illustrated through RStudio. For the average error percentage between ENVI-derived VIs and algorithm-derived VIs, the individual error percentage was calculated, and this individual error % was then averaged (Equation 1)).


(1) $$\text{Individual error }\%=|\frac{A-B}{A}|\times 100\%$$

Where “*A*” is the ENVI-derived VI value and “*B*” is the algorithm-derived VI value.

## 3 Results

### 3.1 Variation of VIs among the accessions

A wide range of variation was observed within the different accessions of soybeans in terms of all the VIs (**Table 2**). In the VC stage, the NDVI value ranged from 0.68 to 0.84 with an average of 0.77, the PRI value ranged from 0.00 to −0.05 with an average of 0.02, the ARI value ranged from −0.84 to 0.85 with an average of −0.27, and the CRI value ranged from 2.79 to 9.78 with an average of 5.00. Among the VIs, PRI had the least variation observed within the accessions, with standard deviation (*SD*) being the least 0.01, and CRI had the highest variation among the accessions, having the highest *SD* of 1.06. A similar result in variation of VIs was observed in the V1 stage as well. The NDVI value here ranged from 0.60 to 0.83 with an average of 0.75, PRI from −0.03 to 0.04 with an average of 0.01, ARI from −0.74 to 0.84 with an average of −0.11 and CRI from 2.78 to 8.76 with an average of 4.93. The CRI here also showed the highest variation in the value with an *SD* of 1.04, and PRI had the least variation with an *SD* of 0.01. Student’s t-test revealed that within the VC stage and V1 stage, there was a significant difference (*p <* 0.05) between NDVI, PRI, and ARI. However, CRI did not show such difference when the comparison was made between these two stages. These findings revealed that the VIs are cultivar-dependent traits. There was a wide variation in VIs among different accessions of soybeans.

**Table 2:** Variation of VIs in soybean accessions among VC and V1 stage.

Stages	Accessions	Vegetation indices			
		NDVI	PRI	ARI	CRI
VC stage	Range (*n* = 258)	0.68–0.84	0.00–0.05	−0.84 to 0.85	2.79–9.78
	Mean ± *SD* (*n* = 258)	0.77 ± 0.03	0.02 ± 0.01	−0.27 ± 0.29	5.00 ± 1.06
	Williams 82 (check)	0.78	0.03	−0.63	4.22
	Sehwa (check)	0.77	0.03	−0.46	3.44
	Hosim2 (check)	0.79	0.04	−0.58	4.22
	Seonpung (check)	0.78	0.04	−0.64	4.16
	Pungsannamul (check)	0.77	0.03	−0.47	4.93
V1 stage	Range (*n* = 258)	0.60–0.83	−0.03 to 0.04	−0.74 to 0.84	2.78–8.76
	Mean ± *SD* (*n* = 258)	0.75 ± 0.04	0.01 ± 0.01	−0.11 ± 0.28	4.93 ± 1.04
	Williams 82 (check)	0.78	0.01	0.01	5.45
	Sehwa (check)	0.76	0.02	−0.11	5
	Hosim2 (check)	0.76	0.02	−0.1	5.24
	Seonpung (check)	0.74	0.02	0.03	5.13
	Pungsannamul (check)	0.76	0.02	−0.02	6.44
T-test between VC and V1 stage		***	***	***	NS

*SD*, standard deviation; *n*, number of accessions; NDVI, normalized difference vegetative index; PRI, photochemical reflectance index; ARI, anthocyanin reflectance index; CRI, carotenoid reflectance index. ***indicates significance at *p* ≤ 0.001, and NS indicates non-significant.

### 3.2 Comparison of VIs based on ROI size and ROI position in leaf

#### 3.2.1 Comparison of VIs based on ROI size

**Figure 3:**
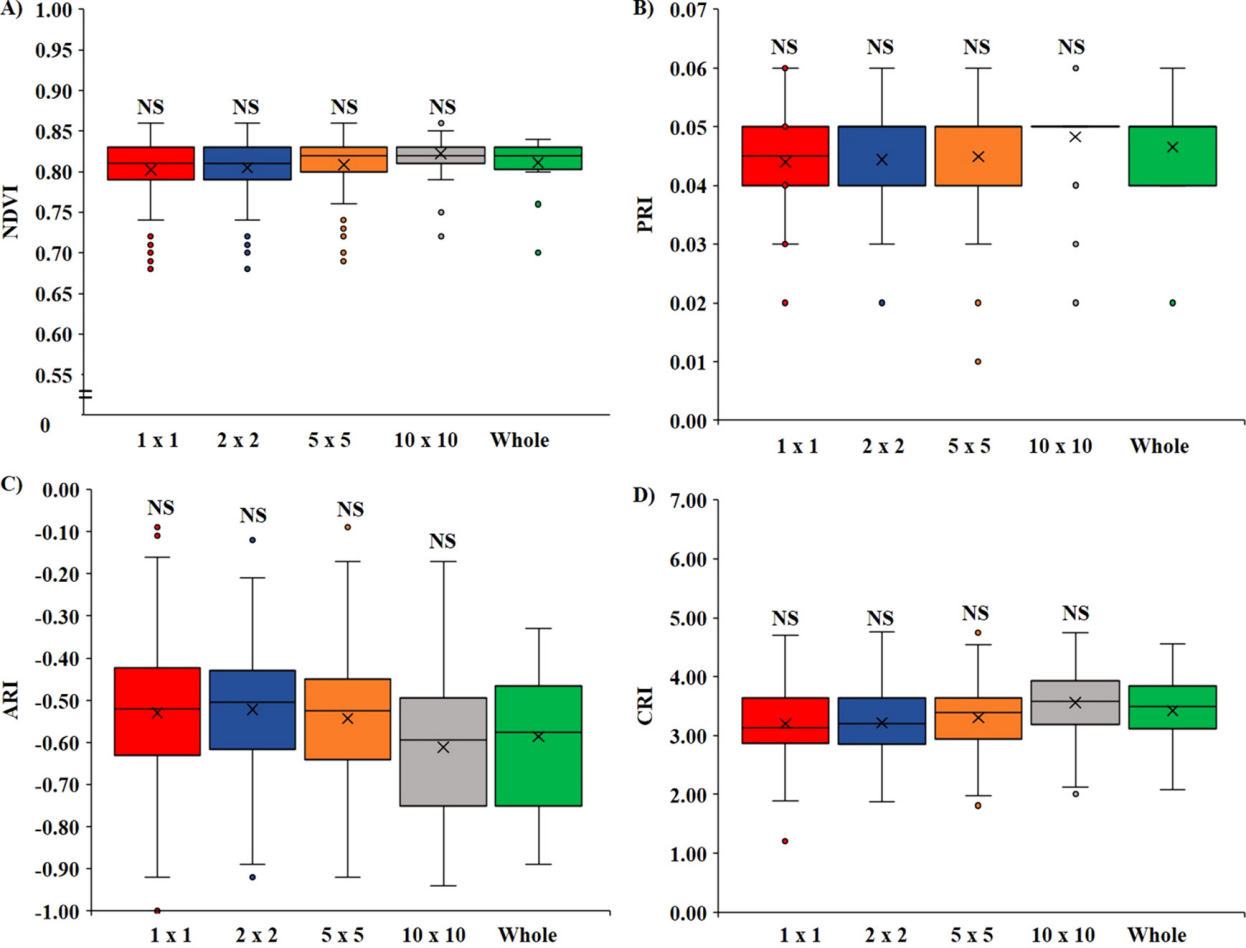
Variation of VIs based on the pixel size of ROI. **(A)** Variation in NDVI, **(B)** variation in PRI, **(C)** variation in ARI, and **(D)** variation in CRI. (NS, non-significant at *p <* 0.05).

The effect on the VIs when there was variation in the pixel size of the ROI is illustrated in **Figure 3**. It was observed that for all the VIs calculated: NDVI (**Figure 3A**), PRI (**Figure 3B**), ARI (**Figure 3C**), and CRI (**Figure 3D**), there were no significant differences (*p* < 0.05) between the ROI size. All the VIs were statistically at par when compared among the 1 × 1, 2 × 2, 5 × 5, and 10 × 10 pixel sizes with whole leaves annotated. However, it was also noted that with the increase in the size of ROI, the *SD* decreased, which means that choosing a larger pixel size would minimize the error difference within the replication.

#### 3.2.2 Comparison of VIs based on ROI position

**Figure 4:**
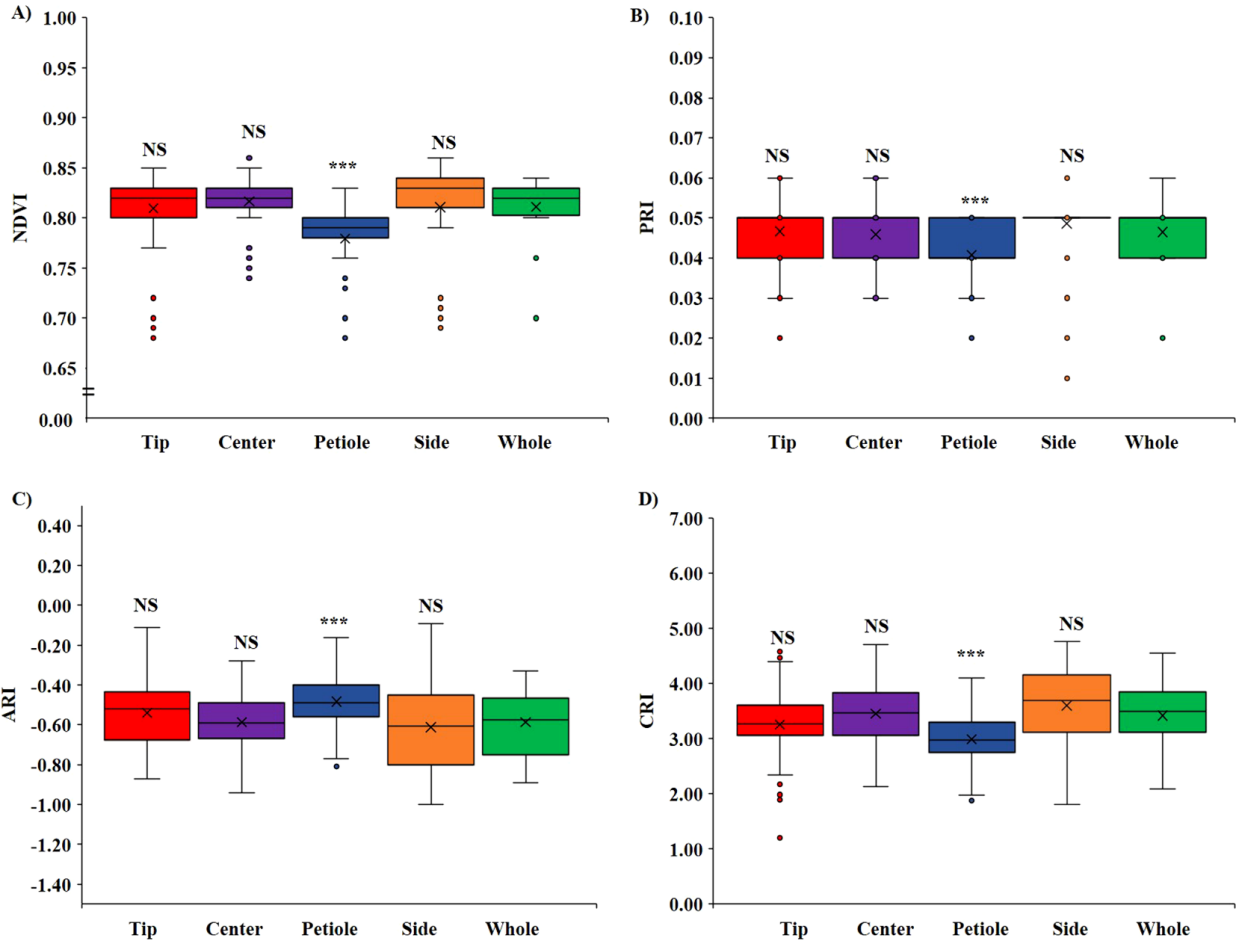
Variation of VIs based on ROI position. **(A)** Variation in NDVI, **(B)** variation in PRI, **(C)** variation in ARI, and **(D)** variation in CRI. (NS, non-significant at *p* < 0.05 and *** = significant at *p* < 0.0001).

**Figure 5:**
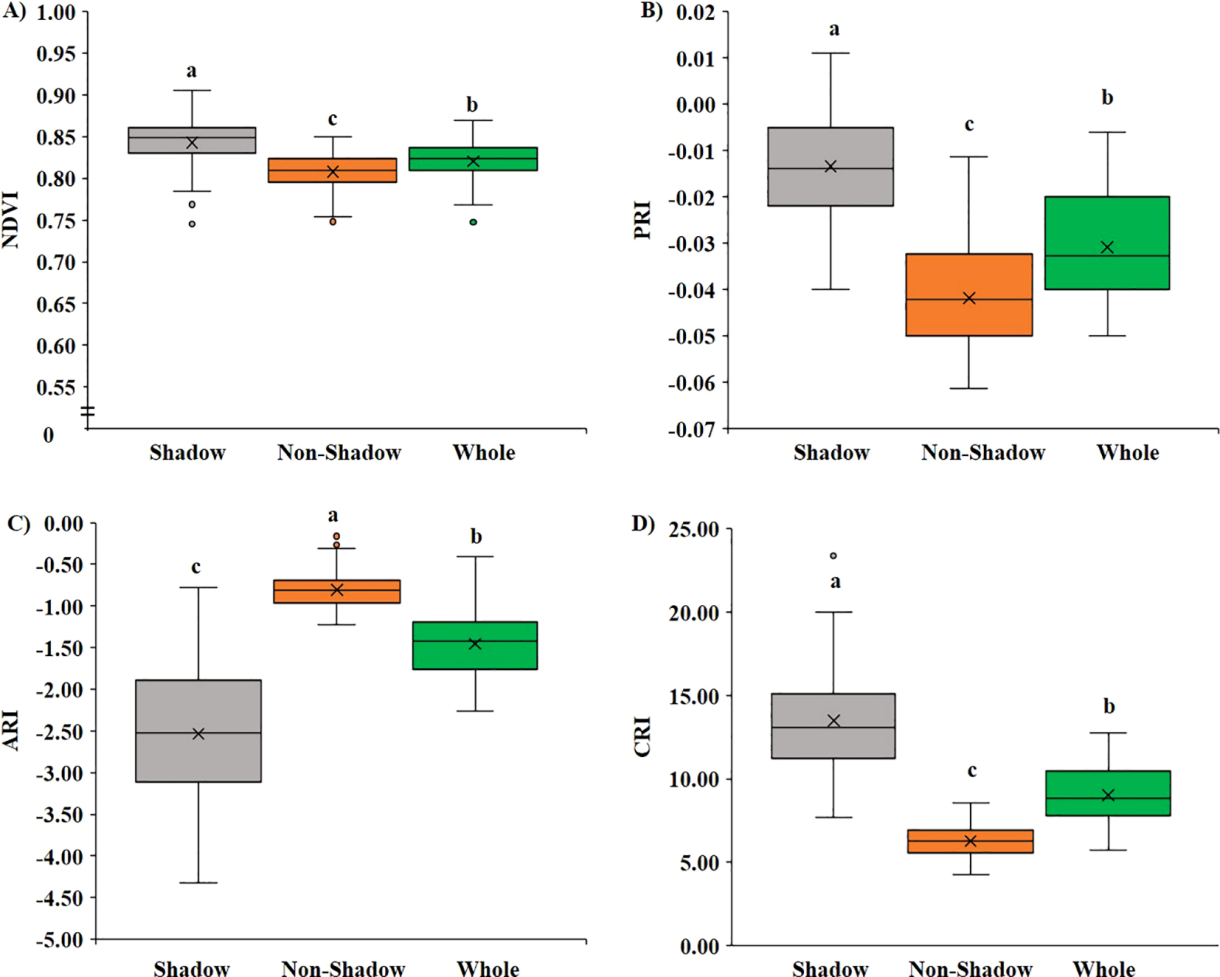
Variation of VIs based on shadowed and non-shadowed regions. **(A)** Variation in NDVI, **(B)** variation in PRI, **(C)** variation in ARI, and **(D)** variation in CRI.

Based on the ROI position on the leaf, we compared two regions; first, we compared VIs when ROIs were on different positions on the same leaf, that is, tip, center, petiole, and side (**Figure 4**), and second, we compared VIs among shaded and non-shaded part of the leaf (**Figure 5**). No significant difference (*p <* 0.05) was observed in all four VIs when a comparison was made between the tip, center, and side of the leaf with the whole leaf annotated. However, the ROI selection on the petiole part had significantly lower (*p <* 0.05) value of NDVI (**Figure 4A**), PRI (**Figure 4B**), and CRI (**Figure 4D**) and higher value of ARI (**Figure 4C**) compared with whole leaf annotation. On average, NDVI decreased significantly by 2.6%, PRI by 12.4%, and CRI by 12.7%, and the ARI increased significantly by 17.6% when the petiole region was annotated compared with the whole leaf.

Similarly, upon investigating the difference in VIs in the shaded and non-shaded part compared with the whole leaf, it was found that the shaded part showed significantly higher (*p <* 0.05) NDVI (**Figure 5A**), PRI (**Figure 5B**), and CRI (**Figure 5D**) and significantly lower (*p <* 0.05) ARI (**Figure 5C**) when compared to both the non-shaded region and whole leaf. In the shaded region, on average, the NDVI value increased significantly by 2.7%, PRI by 56.5%, CRI by 49.8%, and ARI decreased significantly by 74.1%.

### 3.3 Development of automatic ROI selection

The results indicated that there was no variation in VIs regardless of the size of the selected ROI. Similarly, the position of the ROI on the leaf, excluding the shaded and petiole regions, did not significantly affect the VIs. During our study of the variation of VIs on these accessions, we found that the manual selection of ROI and calculation of VIs selecting specific wavebands was tedious and time consuming. So, based on these findings, a Python algorithm was created to select ROI automatically. It was ensured that the ROI selected through the algorithm fell on the center of the leaf. By defining the greenness value, the shaded region (**Figure 2B**) was removed and the center of the largest leaf area was chosen for annotating the ROI. Furthermore, VIs from these selected ROIs were also calculated. As mentioned earlier two different thresholding methods were used for segmenting the image. However, both of these thresholding methods gave the same qualitative result (**Supplementary Figure S3**). The ROI coordinates value (203, 213, 268, and 278) was also obtained to be the same for this representative image, indicating that these thresholding methods did not influence much. The image obtained after the removal of shady and pale parts (**Figure 2B**) has only two-color constraints: black or green, so a simple thresholding method can easily differentiate the image segmentation.

### 3.4 Demonstration of the algorithm with example

A demonstration of the algorithm is presented in **Supplementary Video S1**. The initial step involves inputting the RGB image location and the spectral file location in the code. Once the program is executed, it first displays the image with the defined greenness level, followed by the thresholded image with the highest contour area. Finally, it shows the image with the ROI. This ROI is then used to calculate the VIs. Finally, in the plot window and result window, the figure spectral image, the graph of the mean spectrum, and figures of the selected VIs are displayed along with the value of the VIs of the selected ROI.

### 3.5 Comparison of ENVI-derived VIs with algorithm-derived VIs

**Figure 6:**
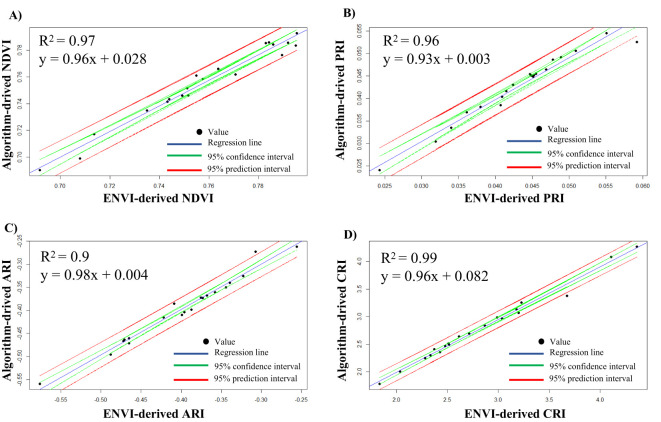
Regression plot for ENVI-derived vegetative indices (VIs) with algorithm-derived VIs. **(A)** Plot for NDVI, **(B)** plot for PRI, **(C)** plot for ARI, and **(D)** plot for CRI.

To compare the results obtained from the automated ROI selection and VIs calculation with ENVI-derived values, we illustrated a linear regression plot within a 95% confidence interval and 95% prediction interval along with a correlation coefficient (*R* ^2^) (**Figure 6**). The values for all the VIs were concentrated within the 95% confidence interval, meaning high precision of the estimated values through the algorithm. The concentrated values within these intervals also suggest low measurement error and a strong relation between ENVI-derived values and algorithm-derived values. Furthermore, a very high *R* ^2^ value > 0.95 was observed in all the VIs (**Figures 6A-D**), the lowest being 0.96 for PRI (**Figure 6B**) and the highest being 0.99 for CRI (**Figure 6D**). Likewise, the slope of the line in all instances was near 1. It was observed that most of the data points of the VIs were closely fitted within the regression line. The 95% confidence interval (green lines) is the range in which the mean value of the algorithm-derived VIs is expected to fall for a given software-derived value (ENVI). The relatively narrow spacing of the confidence interval also suggests a good conformity of the algorithm-derived values. Similarly, the 95% prediction interval (red lines) represents the range in which the VIs are expected to fall within a 95% certainty. This interval is wider than the confidence interval since it provides a range within which 95% of the new observation will fall. However, there are some outliers which represents the cases where the algorithm-derived VIs slightly deviates from the ENVI-derived VIs. This might be due to the fact that the ROI position in algorithm and ENVI might not be exactly on the same place which might have resulted in slight deviation of the VIs values. Noisy data or quality of data might also sometime cause the deviation of the VIs among the algorithm and ENVI calculated VIs. All these results suggest a good conformity between algorithm-derived VIs and ENVI-derived VIs. For an in-depth analysis of the VIs, we have provided the ENVI-derived and algorithm-derived VIs for 20 representative images in **Supplementary Table S1**.

To get more insight into the ENVI-derived VIs and algorithm-derived VIs, we calculated the average error percentage (**Table 3**). It was observed that very low error percentages were observed among all the calculated VIs. The errors for all measurements were below 2.5%, with NDVI having the lowest error at 0.54% and ARI having the highest error at 2.12%. Likewise, we also compared the average time taken for the analysis of a single image in ENVI software and through the algorithm (**Supplementary Table S2**). A significant decrease in time for the analysis was noted. In ENVI, it took us 128 ± 6 s to annotate and calculate the four VIs, whereas this time was significantly reduced to 12 ± 1 s for the same analysis through the algorithm. The algorithm-based analysis was more than ten times faster than the manual ROI selection and band selection for VIs in ENVI.

**Table 3:** Average error for ENVI-derived vegetative indices (VIs) compared with algorithm-derived VIs.

VIs	NDVI	PRI	ARI	CRI
Error %	0.54%	1.87%	2.12%	1.77%

## 4 Discussions

VIs are broadly used as a major parameter for the qualitative and quantitative assessment of the crops. However, the variation of VIs among different accessions of legumes is still an unresearched area. In this study, we explored variations of VIs in diverse soybean accessions, focusing on aspects like leaf greenness, light use efficiency, and leaf pigments. A total of 258 cultivated soybean accessions were evaluated based on four major VIs: NDVI, PRI, ARI, and CRI. In the VC stage, the variation in NDVI ranged from 0.68 to 0.84, PRI from 0.00 to 0.05, ARI from −0.84 to 0.85, and CRI from 2.79 to 9.78. Similar variation was observed in the V1 stage as well, where NDVI varied from 0.60 to 0.83, PRI from −0.03 to 0.04, ARI from −0.74 to 0.84, and CRI from 2.78 to 8.76. A significant difference among these VIs was also observed between these two growth stages, except for CRI. The variation of VIs among the different accessions of the crop can be used to predict the crop growth condition and vigor and generate the yield map based on the variation in the critical growth stage of the crop (Hatfield and Prueger, 2010; Li et al., 2023). VI is derived as a mathematical calculation combining the different wavelengths from the reflectance, which has led to quantifying different agronomic traits like nutrient content, stress condition, growth pattern, and overall plant health (Hatfield et al., 2008; Tayade et al., 2022). It was found that VIs have different patterns of variations among different crops due to their differences in morphology, canopy structure, and photosynthetic pigment concentration. It was reported that the VIs varies depending on the type of crop (Gitelson et al., 2003), genotype (Cabrera-Bosquet et al., 2011), growth stage (Farias et al., 2023), biotic (Römer et al., 2012), and abiotic (Mahlein et al., 2013) stress conditions. In a research carried out by Hatfield and Prueger (2010), it was observed that among the four selected crops (corn, soybean, wheat, and canola), variations in VIs were identified in corn and soybean over different growth seasons, and these differences were attributed to the specific growth stages and VIs of each crop. The study also found that crops with denser canopies and higher biomass tend to increase VIs like NDVI. The variation observed within the soybean accessions could be an essential tool for the genomic selection of soybean cultivars. A study by Rutkoski et al. (2016) used 557 lines of wheat for the selection of high-yielding cultivars based on VIs along with canopy temperature. Another study by Ranđelović et al. (2020) used a variation of VIs in 66 soybean genotypes as predictors for a machine learning model to predict the planting density in soybean.

Accurate calculation of VIs depends on the precise selection of the ROI within a leaf. Similarly, when measuring the VIs, a consistent standard is required because differences in the spectral spectrum and characteristics occur depending on the spatial scale and method of the image acquisition process (Ryu et al., 2018). Based on HSI, our previous research was focused on the evaluation of bacterial disease prediction (Lay et al., 2023), assessment of disease infection (Then et al., 2023), selection of waterlogging tolerant and susceptible soybean cultivars (Kim et al., 2021), and comparison of bentazone-tolerant and susceptible soybean cultivars (Ali et al., 2022) but there persisted a degree of ambiguity regarding the selection of ROI in the spectral images obtained. We were uncertain about selecting the appropriate pixel size when establishing an ROI, particularly concerning VIs within the same leaf when annotating different leaf positions. Furthermore, during our study, we also found that the process of ROI selection and calculation of VIs in ENVI software was time consuming and tedious. The user needs to select the ROI in the leaf manually, and for the calculation of the VIs every time, the wavelength associated with the particular VIs needs to be selected manually. As HTP aims at faster and more accurate data acquisition methods, we were lagging due to this tedious wavelength and ROI selection process in each image. Thus, we created a Python algorithm to automatically define the ROI in such a way that the ROI falls on the center of the leaf with no shaded part. Likewise, if the user finds the automatic selection of ROI inconvenient, they can manually select their own ROI also, and the calculation of VIs is then done automatically without needing to select a specific wavelength every time.

In our study, a significant increase in VIs was observed for shaded regions. Rasmussen et al. (2016) found a similar difference in VIs value between the brightly captured part and dark acquired image. Zhang et al. (2015) findings indicate that shadow has an impact on individual narrow bands of a VI and influences the vegetation parameters. Leaves in these shaded regions have greater reflectance in the red and blue regions, where pigments absorb light, resulting in higher VIs (Zhang et al., 2015; Hallik et al., 2017). HSI has a high spatial resolution, with many pixels per leaf (Tayade et al., 2022); when VIs are calculated in HSI, they are based on the spectral reflectance properties of the vegetation, not the spatial pixel size, which might be the reason for the non-significant difference in VIs values based on the ROI size. The VIs are calculated based on the spectral properties of the photosynthetically active vegetation components (https://www.nv5geospatialsoftware.com/docs/VegetationIndices.html, accessed on 9 May 2024). The petiole of the plant has a lower amount of photosynthetic pigments like chlorophyll content, lower chloroplast number, and decreased ratio of chlorophyll a/b compared with whole leaf (Sun et al., 2021). Thus, with a difference in photosynthetically active vegetation components, the VIs calculated in the petiole region showed a significant difference with the VIs on the leaf. Likewise, we created a Python algorithm for automatic ROI selection and VIs calculation. A significant reduction in time for VIs calculation time was observed in algorithm-based analysis per image. As ROI selection is done automatically and the formula for each VIs is already written, we just need to import the reflectance file and RGB image and run the algorithm (**Supplementary Figure S4**). Hyperspectral imaging in field conditions can be challenging due to uneven lighting and the presence of many plants. The field environment has many shadows and the leaf angle also differs in such case (Liu et al., 2020a). To address the issue of dynamic field lighting, which causes significant changes in shaded and non-shaded regions of plants, we provided users with an option to define their own ROI. This allows users to optimize the ROIs themselves and extract VIs based on their selected ROIs. This approach not only addresses lighting issues (shaded and non-shaded regions) but also lets users choose their target plants in the field. Furthermore, the ROI optimized by the users themselves can be used to extract the VIs of matured leaves also or any other stage of leaves. Apart from leaves, VIs of any other plant part can also be calculated automatically once the ROI has been optimized. To generalize the algorithm for crops other than soybean, we analyzed sesame and cowpea for ROI optimization. The qualitative results for ROI optimization in these crops were similar to those for soybean, with ROIs falling at the midpoint of the largest contoured leaf area. **Supplementary Figures S5**, **S6** show these qualitative results. Sesame and cowpea were imaged under natural lighting conditions, further supporting the generalization of the proposed method. The ability to use the method in field conditions and for various crop species increases its potential for broader applications. In this study, we mainly focused on four major VIs. However, other VIs, such as the green leaf index (GLI), simple ratio (SR), modified simple ratio (MSR), structure-insensitive pigment index (SIPI), modified chlorophyll absorption ratio index (MCARI), and many others can also be calculated. Their formulas, based on wavelength, can simply be added to the algorithm. This allows for the calculation of many other VIs in a short time without rewriting formulas repeatedly. These explanations highlight the applicability and generalization of the proposed method in a broader precision agriculture system. Sometimes, when the leaf size is small, the ROI can be plotted outside the leaf region (**Supplementary Figure S7**), which adds to the limitation of the study. In such cases, we can change the height and width of the ROI. As the pixel size does not have a significant difference in VIs, the size of the ROI can be adjusted in the algorithm. Furthermore, to add some limitations to our study, we studied only the upper leaf portion while accessing the variation in the soybean germplasm. However, some findings suggest that crop reflectance properties cannot be solely dependent on the upper leaves. For example, it was found that in wheat, along with the upper leaf, the lower leaf, stems, and spikes also had a significant effect on the canopy reflectance characteristic and the VIs obtained (Li et al., 2015). Furthermore, in case of narrow leaf plant like wheat where flag leaf is usually selected to represent the whole plant for VIs calculation (Liu et al., 2020b), the optimized ROI might fall outside of this flag leaf which adds some limitation to this study. However, the ROI optimization based on user can solve this problem to some extent as the ROI is optimized by the user themselves so that they can choose the ROI to be placed on the flag leaf or the leaf as they desire. Likewise, in this study every sort of preprocessing was done on the RGB images, the spectral image acted only as data source for mean spectrum value for the calculation of VIs. A more comprehensive study is required to integrate the preprocessing in both RGB image and spectral image so that the difference in ROI optimization in between these two image types can be explored.

## 5 Conclusion

Through this study, we found the best position for ROI selection for VIs measurement, that is, the leaf part excluding the petiole and shaded part of the leaf. Likewise, the size of pixels while selecting ROI does not significantly affect the results. However, we suggest selecting a bigger pixel size, which results in a lower *SD* (lessening the errors). Similarly, the variation of VIs on soybean accessions suggests cultivar-dependent VIs. Furthermore, we present an algorithm-based method for ROI selection and VIs calculation for user convenience. This algorithm was correlated with ENVI software-derived values, which showed a higher correlation and high accuracy. Therefore, this comparative analysis aims to pinpoint the most effective ROI parameters for precise and dependable determination of VIs along with easy and automatic way of ROI selection and VIs calculation, which are important for diverse applications in plant science.

## Data Availability

The datasets presented in this study can be found in online repositories. The names of the repository/repositories and accession number(s) can be found in the article/**Supplementary Material**.
