# Supplementary material for: Automatic optimization of regions of interest in hyperspectral images for detecting vegetative indices in soybeans
Source: Front Plant Sci. 2025 Mar 6;16:1511646. doi: 10.3389/fpls.2025.1511646 (PMC11922918; doi:10.3389/fpls.2025.1511646)
Supplement: Supplementary file 1 [file DataSheet1.docx]

**Supplementary material**


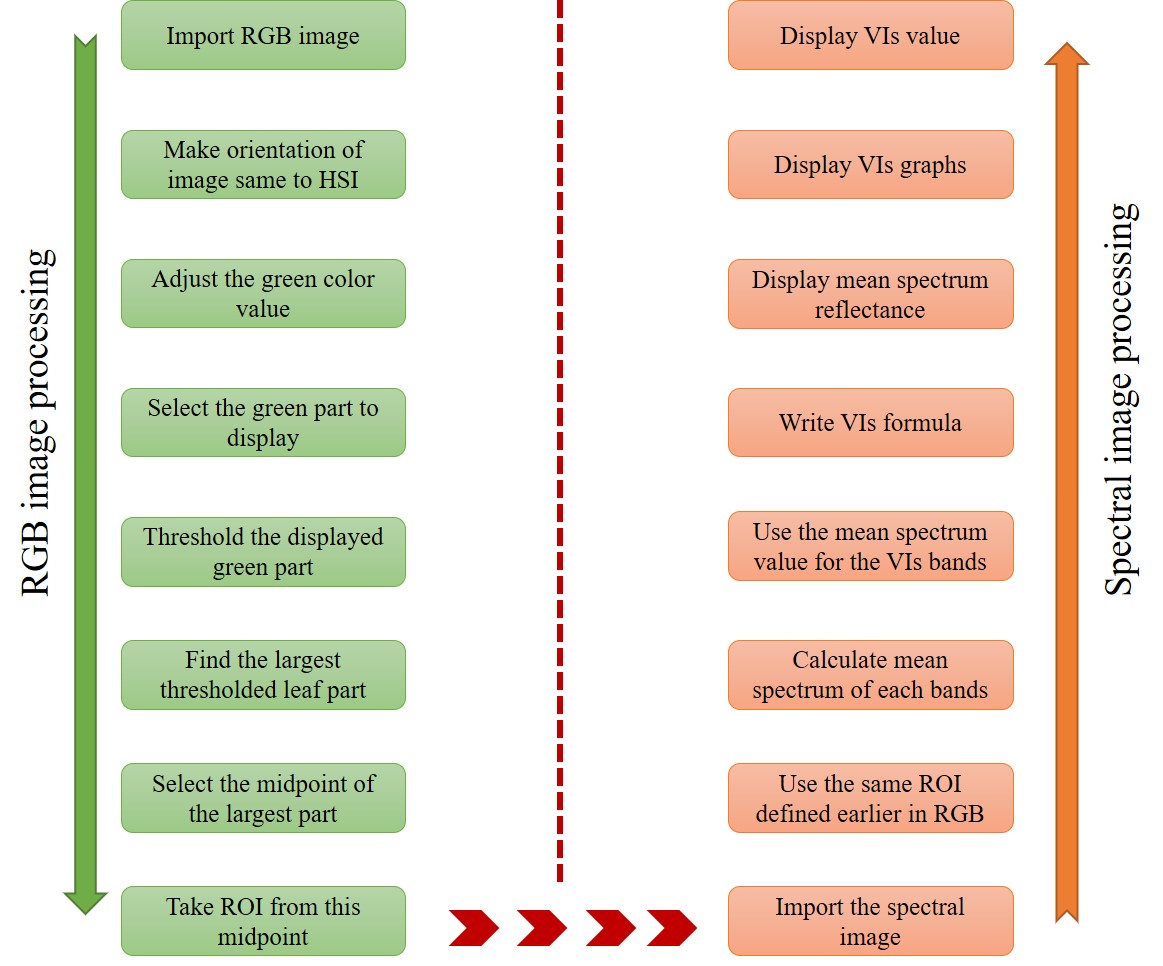


**Figure S1.** Flowchart of the methodology for extraction of ROI and calculation of vegetative indices (VIs) automatically.


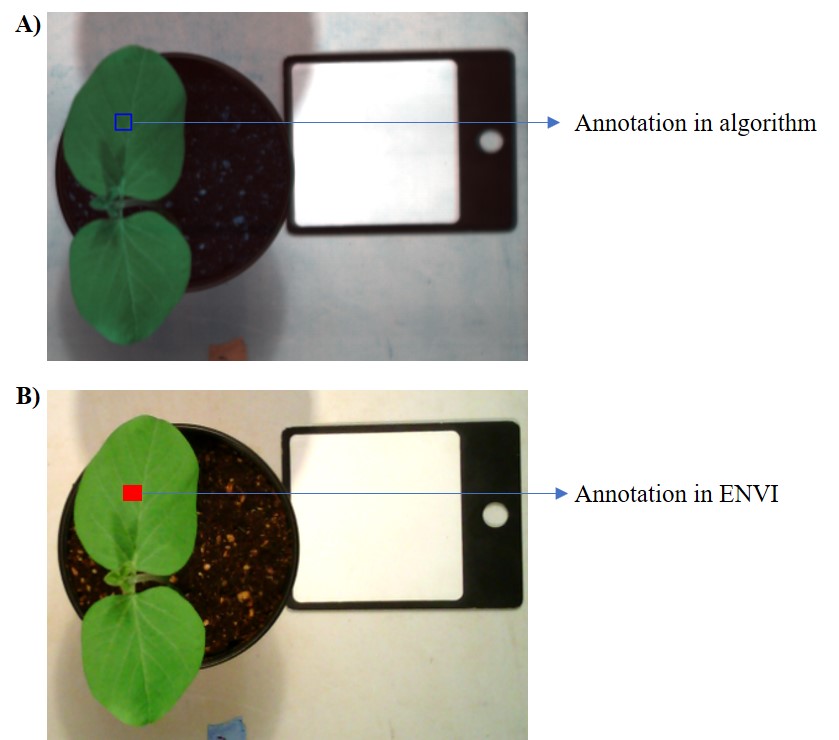


**Figure S2**. Selection of ROI. (A) Automated ROI in algorithm, and (B) manual ROI selection in ENVI.


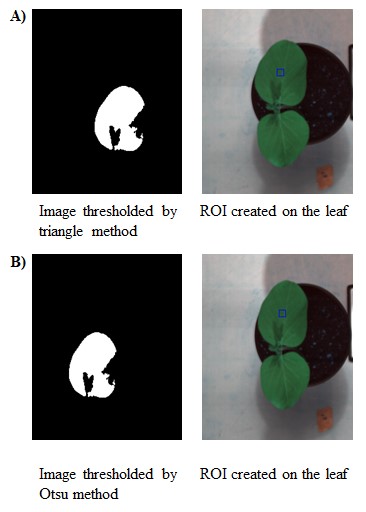


**Figure S3.** Image thresholded by two different methods and their respective ROI position. (A) Image thresholded by triangle method and the ROI position and (B) Image thresholded by Otsu method and the ROI position.


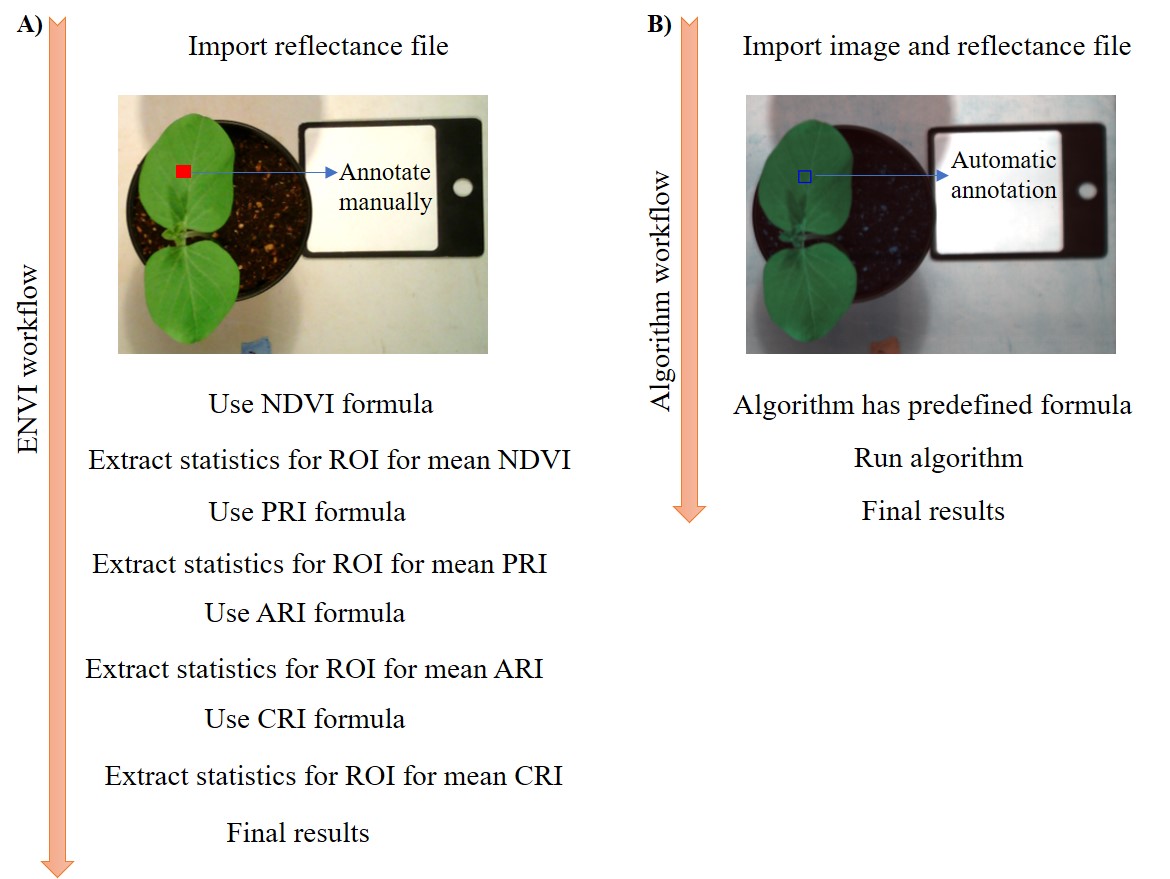


**Figure S4.** Workflow differences in ENVI and algorithm. (A) Analysis process in ENVI and (B) analysis process in algorithm.


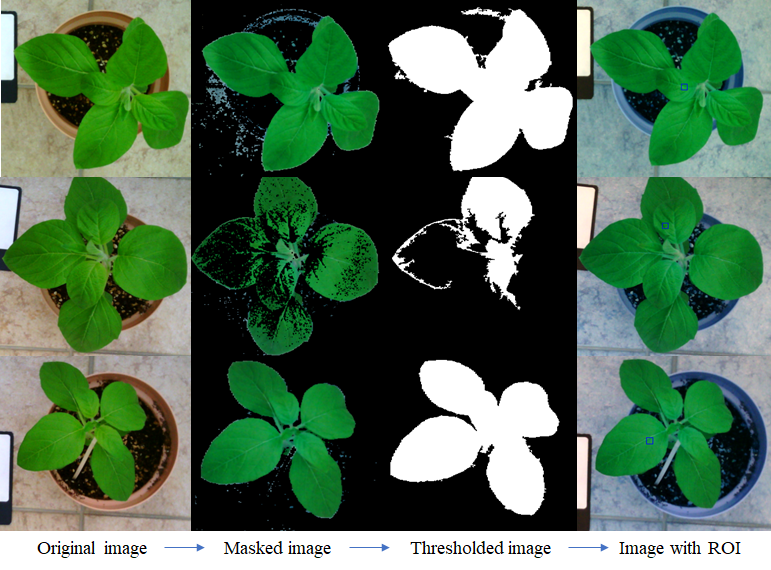


**Figure S5**. Process of ROI optimization in sesame plant leaves.


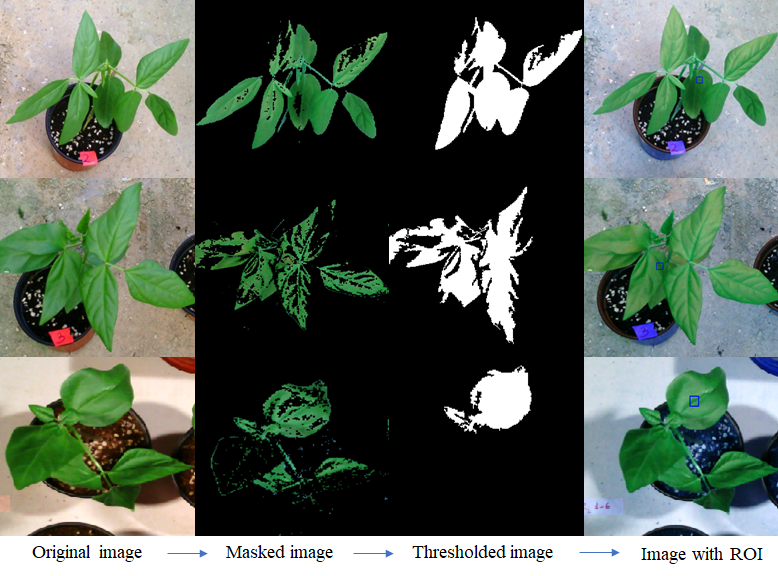


**Figure S6**. Process of ROI optimization in cowpea leaves.


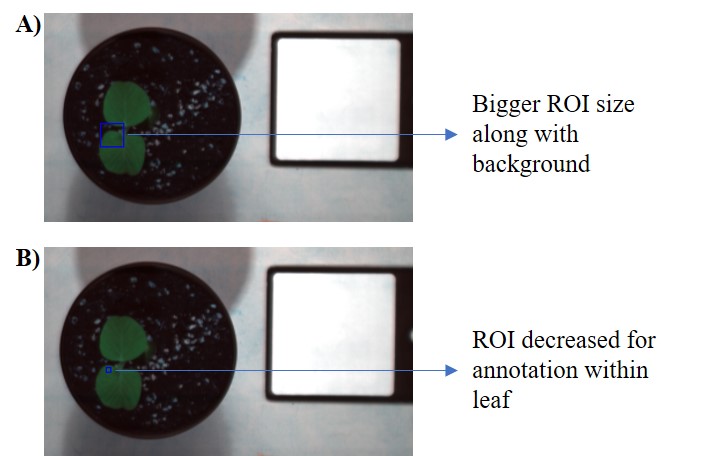


**Figure S7**. ROI selected out of leaf region and within leaf region. (A) Bigger size ROI out of leaf region, and (B) size of ROI decreased to keep it within leaf region.

**Table S1**. ENVI-derived VIs and algorithm-derived VIs.

| ENVI-derived values | | | | Algorithm-derived values | | | |
| --- | --- | --- | --- | --- | --- | --- | --- |
| NDVI | PRI | ARI | CRI | NDVI | PRI | ARI | CRI |
| 0.708 | 0.036 | -0.396 | 2.427 | 0.699 | 0.037 | -0.404 | 2.358 |
| 0.792 | 0.041 | -0.465 | 4.108 | 0.786 | 0.038 | -0.471 | 4.083 |
| 0.758 | 0.045 | -0.488 | 2.989 | 0.759 | 0.045 | -0.496 | 2.985 |
| 0.784 | 0.051 | -0.471 | 3.228 | 0.786 | 0.051 | -0.463 | 3.255 |
| 0.752 | 0.046 | -0.576 | 3.035 | 0.746 | 0.045 | -0.560 | 2.969 |
| 0.786 | 0.055 | -0.465 | 3.178 | 0.784 | 0.055 | -0.460 | 3.134 |
| 0.771 | 0.032 | -0.409 | 3.200 | 0.762 | 0.030 | -0.386 | 3.069 |
| 0.796 | 0.034 | -0.399 | 4.362 | 0.793 | 0.033 | -0.410 | 4.268 |
| 0.735 | 0.038 | -0.368 | 2.715 | 0.735 | 0.038 | -0.368 | 2.693 |
| 0.795 | 0.041 | -0.376 | 3.676 | 0.784 | 0.040 | -0.372 | 3.374 |
| 0.783 | 0.045 | -0.344 | 2.868 | 0.785 | 0.045 | -0.350 | 2.835 |
| 0.744 | 0.042 | -0.256 | 2.280 | 0.743 | 0.042 | -0.262 | 2.248 |
| 0.749 | 0.049 | -0.323 | 1.835 | 0.746 | 0.049 | -0.325 | 1.781 |
| 0.755 | 0.048 | -0.387 | 2.511 | 0.761 | 0.049 | -0.398 | 2.511 |
| 0.692 | 0.024 | -0.358 | 2.515 | 0.690 | 0.024 | -0.361 | 2.492 |
| 0.714 | 0.042 | -0.340 | 2.371 | 0.717 | 0.043 | -0.340 | 2.407 |
| 0.751 | 0.045 | -0.472 | 2.478 | 0.751 | 0.045 | -0.467 | 2.465 |
| 0.743 | 0.047 | -0.422 | 2.334 | 0.741 | 0.046 | -0.415 | 2.298 |
| 0.764 | 0.045 | -0.373 | 2.615 | 0.766 | 0.045 | -0.373 | 2.638 |
| 0.790 | 0.059 | -0.307 | 2.034 | 0.776 | 0.053 | -0.272 | 2.008 |

**Table S2.** Time taken for analysis between ENVI and algorithm.

|  | ENVI (seconds) | Algorithm (seconds) |
| --- | --- | --- |
|  | 131 | 12.15 |
|  | 129 | 11.83 |
|  | 134 | 11.32 |
|  | 141 | 11.26 |
|  | 122 | 12.24 |
|  | 124 | 10.45 |
|  | 139 | 12.23 |
|  | 128 | 12.12 |
|  | 124 | 12.11 |
|  | 134 | 14.93 |
|  | 126 | 11.9 |
|  | 123 | 12.03 |
|  | 118 | 13.94 |
|  | 120 | 10.87 |
|  | 137 | 11.71 |
|  | 122 | 12.23 |
|  | 123 | 12.64 |
|  | 125 | 11.76 |
|  | 130 | 13.74 |
|  | 131 | 11.44 |
| Mean | 128.05 | 12.15 |
| Standard deviation (SD) | 6.48 | 1.05 |

**Video S1.** Demonstration of the process of analysis in Python environment.
